# The role of direct feedback in improving hand hygiene technique

**DOI:** 10.1186/2047-2994-4-S1-P288

**Published:** 2015-06-16

**Authors:** Á Lehotsky, L Szilágyi, G Wéber, P Róna, R Pethes, P Szerémy, T Haidegger

**Affiliations:** 1 Infection control, CLARITON, Budapest, Hungary; 2 CLARITON, Budapest, Hungary; 3 Semmelweis University, Budapest, Hungary; 4 Dept. of Control Engineering and IT, CLARITON, Budapest, Hungary

## Introduction

Electronic equipment helps to improve hygiene-related behavior giving instant feedback concerning the quality of hand hygiene performance.

## Objectives

This study was designed and conducted to analyze the lasting effects of instantaneous objective feedback to the staff regarding the hand rubbing technique.

## Methods

Our study involved 9 wards from 3 hospitals and was based on Hand-in-Scan Medical Trainer device that employs digital imaging and computer image processing to identify all missed areas on the hand, and display them unambiguously to the healthcare worker. The equipment was present for 3-4 weeks. Participants were identified with RFID card and had the opportunity to use the device once every day. A total of 572 measurements were performed involving 129 healthcare workers.

## Results

The surface of hand was divided into 20 regions for clustered evaluation. For each measurement, the number of missed spots in each region was compiled by an expert based on recorded images. Performances were classified as correct or erroneous based on the total number of mistakes. Our study showed that average number of mistakes dropped significantly between the first and the second hand rubbing occasion. Another relevant improvement was detected when we compared the outcome of the 2nd–4th occasions (clustered) with the 5th–10th ones (clustered). Considering all records, the baseline value 1.5 of average number of mistakes dropped to 0.35 after five hand rubbing events.

## Conclusion

Direct feedback of Hand-in-Scan device supports correcting wrong practices and eliminating erroneous habits. During 3–4-week-long follow-up studies, the average number of missed spots on the hands reduced significantly at every site.

## Disclosure of interest

None declared.

